# Protoblock - A biological standard for formalin fixed samples

**DOI:** 10.1186/s40168-020-00901-1

**Published:** 2020-08-22

**Authors:** Yensi Flores Bueso, Sidney P. Walker, Glenn Hogan, Marcus J. Claesson, Mark Tangney

**Affiliations:** 1 CancerResearch@UCC, University College Cork, Cork, Ireland; 2 SynBioCentre, University College Cork, Cork, Ireland; 3 APC Microbiome Ireland, University College Cork, Cork, Ireland; 4 School of Microbiology, University College Cork, Cork, Ireland

**Keywords:** FFPE, Microbiome, PCR, DNA, Bacteria, Microscopy

## Abstract

**Background:**

Formalin-fixed, paraffin-embedded (FFPE) tissue is the gold standard in pathology tissue storage, representing the largest collections of patient material. Their reliable use for DNA analyses could open a trove of potential samples for research and are currently being recognised as a viable source material for bacterial analysis. There are several key features which limit bacterial-related data generation from this material: (i) DNA damage inherent to the fixing process, (ii) low bacterial biomass that increases the vulnerability to contamination and exacerbates the host DNA effects and (iii) lack of suitable DNA extraction methods, leading to data bias. The development and systematic use of reliable standards is a key priority for microbiome research. More than perhaps any other sample type, FFPE material urgently requires the development of standards to ensure the validity of results and to promote reproducibility.

**Results:**

To address these limitations and concerns, we have developed the Protoblock as a biological standard for FFPE tissue-based research and method optimisation. This is a novel system designed to generate bespoke mock FFPE ‘blocks’ with a cell content that is user-defined and which undergoes the same treatment conditions as clinical FFPE tissues. The ‘Protoblock’ features a mix of formalin-fixed cells, of known number, embedded in an agar matrix which is solidified to form a defined shape that is paraffin embedded.

The contents of various Protoblocks populated with mammalian and bacterial cells were verified by microscopy. The quantity and condition of DNA purified from blocks was evaluated by qPCR, 16S rRNA gene amplicon sequencing and whole genome sequencing. These analyses validated the capability of the Protoblock system to determine the extent to which each of the three stated confounding features impacts on eventual analysis of cellular DNA present in FFPE samples.

**Conclusion:**

The Protoblock provides a representation of biological material after FFPE treatment. Use of this standard will greatly assist the stratification of biological variations detected into those legitimately resulting from experimental conditions, and those that are artefacts of the processed nature of the samples, thus enabling users to relate the outputs of laboratory analyses to reality.

Video Abstract

## Background

Increased sequencing capabilities have driven progress in the study of the human microbiome [1–3], and distinct microbial profiles have been reported in body sites previously thought of as sterile (although many are potentially influenced by environmental contamination) [4–9]. These discoveries have steered a higher demand for patient samples, availability of which can be highly constrained when sampling from body sites that involve invasive sampling procedures [10, 11].

In an attempt to satisfy this demand, the use of formalin-fixed paraffin-embedded tissue (FFPE) has been explored for microbiome research [12–19]. FFPE tissue is the gold standard for pathology tissue storage and thus represents the largest collection of available patient material [20–22]. The availability of this material has been vital for progress in human genomics, and numerous sequencing workflows have been designed to enable use of, or are based upon, these samples [23–28]. The use of FFPE tissue for microbiome research could open access to large sample cohorts (guaranteeing statistical power), accompanied by a clear clinical history and histology reports. However, FFPE samples carry several limitations and considerations must be taken into account before their reliable use in microbiome research. Investigations into the quality of DNA from human FFPE samples have revealed that factors in the processing and storage (e.g. length of exposure to formalin, pH of formalin and sample storage time) negatively impact the integrity of nucleic acids and the efficacy of their downstream analyses [29, 30]. Relevant to microbiome research, unique factors to consider in quality control of FFPE samples are:

1. *Low biomass* renders samples extremely susceptible to the high burden of contaminants to which they are exposed during the non-sterile FFPE processing [31]. Additionally, it aggravates the influence of host DNA, rendering samples ineffective for whole genome sequencing (WGS) and introducing PCR bias to 16S rRNA gene amplicon sequencing [32].
2. *FFPE causes DNA damage*, in the form of crosslinks, DNA fragmentation and sequence alterations [33]. In this context, 16S rRNA gene amplicon sequencing (V3-V4) necessitates DNA fragments with a length of 460 bp and sequence alterations may lead to false speciation events.
3. *No sample prep methods* available for microbiome study of this sample type. FFPE microbiome studies to date have utilised approaches designed for FFPE human samples, which are suboptimal for this aim [34].

In order for the potential value of increased usage of FFPE samples for metataxonomics/metagenomics to be realised, the necessary workflows, protocols and quality control standards need to be in place [25, 35–37]. Among these, the development and systematic use of biological standards have been recently highlighted as a key priority for microbiome research [38–41]. Given the multiple variables (FFPE processing, storage and DNA isolation process) that directly influence the quantity and quality of DNA recovered from FFPE samples, more than perhaps any other sample type, FFPE tissue urgently requires the development of standards to ensure the validity and reproducibility of results.

A model that serves as a standard for metataxonomic and metagenomic analysis of FFPE samples requires: (1) a defined bacterial and host cell load, (2) exposure to the same treatment as FFPE specimens (fixation and dehydration), (3) a format that resembles FFPE blocks—enabling the same treatment as the source material (sectioning, deparaffinisation). Here presented is the Protoblock, to serve as a biological standard for FFPE samples. The Protoblock is a cell matrix, which can be populated with cell types and numbers as desired, such as to resemble those of the FFPE tissue specimens. It can be integrated in the workflow either at the FFPE processing stage for prospective studies or at the sample prep stage for retrospective studies, allowing the assessment of either workflows, highlighting caveats that must be considered when analysing the sequencing results.

This study describes: (1) the procedures to make the Protoblock and its validation by microscopy and (2) validation of their value as a standard for 16S rRNA gene amplicon sequencing and WGS.

## Results

### Protoblock generation and validation

#### Making the Protoblock

**Fig. 1:**
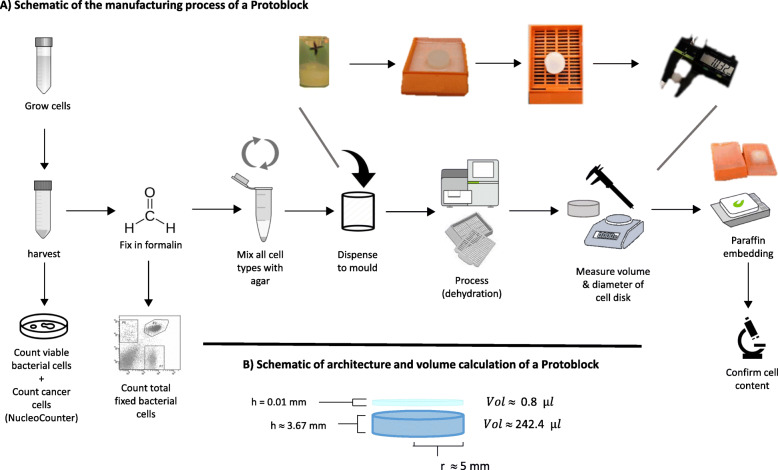
Making a Protoblock. **a** Schematic of the workflow for making a Protoblock described in methods. **b** Schematic of the architecture of a Protoblock, demonstrating average measurements of volume, height and radius

The Protoblock is generated by embedding a known number of fixed cells in an agar matrix that is poured into a mould that renders a defined uniform shape, in this example, a disk. Once the agar solidifies, the blocks are processed as per routine FFPE processing protocols for dehydrating and paraffin embedding and verified by microscopy, see Fig. 1a for procedures used to prepare the Protoblock.

**Fig. 2:**
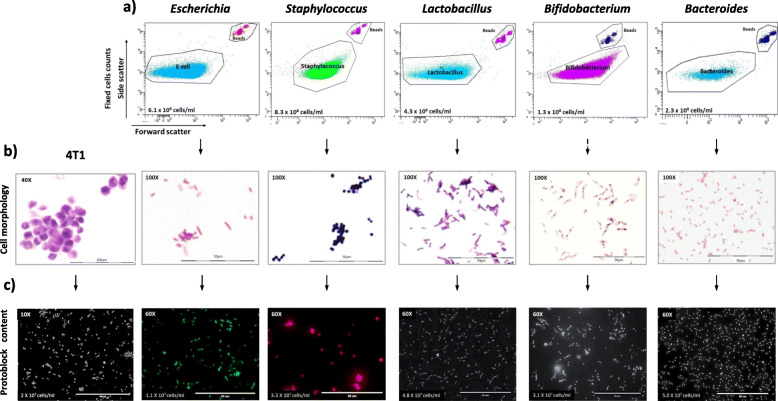
Validation of cell architecture and numbers in a Protoblock. **a** Flow cytometry dot plots measuring the cell density of fixed bacterial suspensions used to make Protoblocks. Events were gated either for SYTOBC+ cells or beads. The averages of 3 reads for 4 populations per cell type are shown here and in Table 1. **b** Light microscopy images confirming cell architecture of Protoblock slides stained with H&E (4T1 cells) or Gram staining (bacteria). **c** Fluorescence microscopy images confirming cell content of Protoblocks. Slides were with α-*E. coli* (green), α-*S. aureus* (red) or DAPI (grey). Counts in Fig. 1 are the average of 20 FOV in 3 × 4 μm slides

To achieve the desired cell numbers, formalin-fixed cell suspensions were counted with FACS (bacteria) or NucleoCounter (4T1) and the volume of the cell suspensions normalised to cell contents (Fig. 2a). The Protoblock radius, height and volume were measured after dehydration. Average measurements for Protoblocks presented here were 4.99 ± 0.15 mm, 3.57 ± 0.24 mm and 245.2 ± 14.2 μl, respectively. A slide’s estimated cell population was calculated by multiplying the cell content per microliter of block by the volume of a 20-μm slide (*x̄* = 1.57 μl, *σ* = 0.098 μl) or 4-μm slide (*x̄* = 0.39 μl, *σ* = 0.02 μl), see Fig. 1b.

#### Protoblock validation

**Fig. 3:**
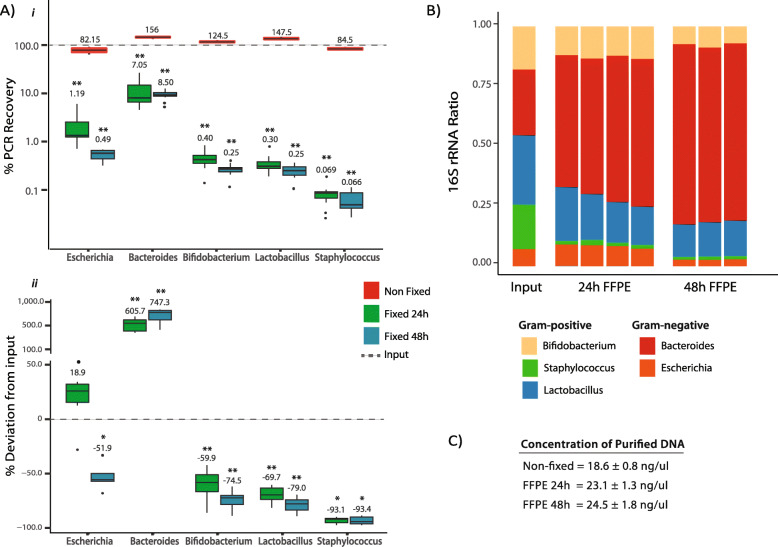
Assessing the recovery of FFPE bacterial DNA by quantitative PCR and 16S rRNA sequencing. **a** Evaluating PCR recovery of FFPE bacterial DNA from Protoblocks fixed for 24 h (green) or 48 h (cyan) and compared with the recovery of paired NF samples (red). (i) % of absolute PCR recovery (% shown above corresponding box). A 2-log fold decrease in recovery is observed for FFPE-treated samples, which was found to be statistically significant in all cases as per 1 sample Wilcoxon signed-rank test. In addition, longer fixation periods lead to a significantly greater reduction in recovery (*p* = 0.04). (ii) % deviation in recovery after compensating for 10-fold loss in recovery. Input = 0 (dotted line). % deviation shown above corresponding box. Significant deviation from input values, even after compensation for 10-fold decrease shown in all FFPE-treated samples. (In all cases, *p* = + < 0.1, * < 0.05, ** < 0.01 and *** < 0.001). **b** Sample composition bar plot of calculated input of bacterial cells added to Protoblock and 16S rRNA gene sequence analysis of Protoblocks fixed for 24 h or 48 h. **c** Average concentration of DNA purified from samples

Protoblocks were populated with cell types and cell loads that provided the best resolution for each experimental aim. Comparable ratios of a mix of 5 bacterial strains and 4T1 cells (in the same order of magnitude ≅ 1 × 10^7^) were aimed for. Estimated cell content was confirmed by immunofluorescence microscopy in blocks containing individual cell types (Fig. 2c) and mixed cell content (sFigure 1). Cell wall/membrane integrity was assessed by Gram (bacteria) or haematoxylin & eosin (H&E) staining (4T1 cells), see Fig. 2b. The calculated and confirmed contents for each Protoblock are specified in Fig. 3 and sTable 1.

### Protoblock as a standard for assessing and optimising 16S amplicon sequencing and WGS workflows of FFPE samples

#### A. Assessing the efficacy of currently used methods for purifying bacterial FFPE DNA

Total DNA was extracted from Protoblocks fixed in formalin for 24 or 48 h (10 × 15 μm slides) using the ‘gold standard’ DNA purification method for FFPE samples used in previous FFPE 16S rRNA amplicon gene sequencing studies (QIAGEN FFPE DNA kit). Recovery was determined via qPCR of strain specific ≅ 460-bp DNA fragments (length relevant for 16S rRNA amplicon gene sequencing). As seen in Fig. 3a (i), FFPE-treated samples had at least a 10-fold reduction in the amount of amplifiable DNA, shown to be statistically significant (*p* < 0.001). Although similar amounts of DNA were purified from the samples (Fig. 3c), the PCR readability of DNA is reduced by FFPE treatment, which is aggravated with increasing fixation time. Furthermore, after compensating for the 2-log fold loss of readable DNA, statistically significant under- and over-representation of all 5 genera present was evident, with a clear bias towards Gram-negative (G−) bacteria (*Bacteroides* and *Escherichia*). This was more evident for *Bacteroides* and *Staphylococcus*, which were over- and under-represented by 605% and − 93.1% respectively (Fig. 3a (ii)). This effect was exacerbated by longer fixation periods. Lysis bias was confirmed with 16S rRNA gene amplicon sequencing (Fig. 3b). Altogether, these data indicate that a bacterial lysis mechanism must be incorporated in the workflow for processing of FFPE samples (this is not included in the QIAGEN kit, optimised for human DNA purification) and that for bacterial FFPE DNA, the baseline recovery of 460-bp fragments is ≤ 2-log the input. The results from these tests in Protoblocks were corroborated by FFPE murine tumour models as shown in sFigure 2.

#### B. Assessing the sources of bias

Next, it was examined whether Protoblocks prove useful for investigating aspects that can introduce bias to a PCR or a sequencing run and for exploring strategies that reduce it.

##### Membrane/cell wall disruption

**Fig. 4:**
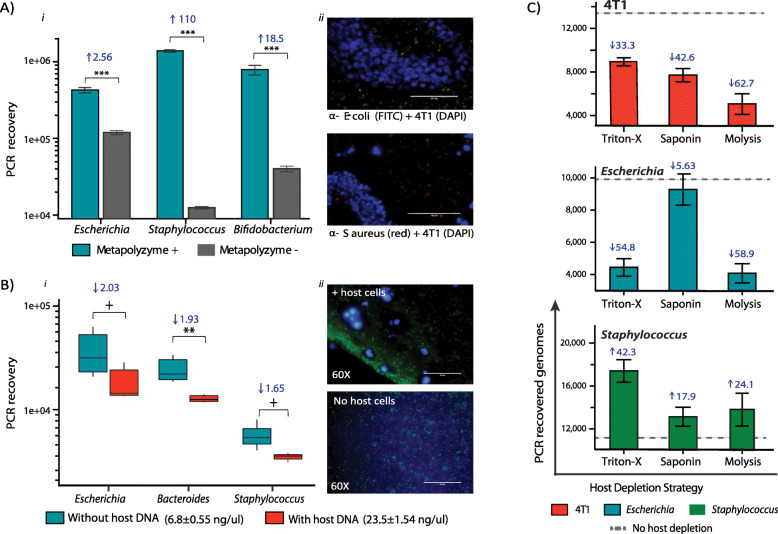
Evaluating bias in sample composition. **a** Metapolyzyme lyses FFPE bacteria. (i) Bar plot showing quantitative PCR DNA recovery after lysis (cyan)/no lysis (grey) with Metapolyzyme. Increase in recovery is shown above each test. For each bar, *n* = 6. Treatment with 100 μg of Metapolyzyme for 4 h markedly increased the recovery of DNA in all tests (*p* < 0.001) as per Wilcoxon signed-rank test. (ii) Immunofluorescence microscopy images of Protoblocks stained with DAPI (blue) for 4T1 cells, α-*E. coli* (green) and α-*S. aureus* (red). Protoblocks were fixed for 24 h. **b** Measuring bias introduced by host DNA. (i) Box plot comparing DNA recovery of bacteria in Protoblocks loaded with (cyan) and without 4T1 cells (orange). Quantitative PCR recovery was normalised to a sample input of 10^6^ cells. For each box, *n* = 6. Protoblocks without 4T1 cells had a higher recovery of all bacteria taxa. Difference of means between tests was measured using a Wilcoxon signed-rank test, for all bacterial taxa. (ii) Immunofluorescence microscopy images of Protoblocks with and without mammalian cells, stained with α-*E. coli* (green) and DAPI (blue) for 4T1 cells. Protoblocks were fixed for 48 h. **c** Testing host DNA depletion strategies. DNA recovery of 4T1 cells (orange), Escherichia (cyan) and Staphylococcus (green) after a 10-min treatment with either Triton-X (0.1%), Saponin (0.1%) or Molysis CM buffer. For each bar, *n* = 3. % increase or decrease in recovery from untreated is shown above each bar. Dotted lines indicate the PCR recovery of samples without host depletion. (In all cases, *p* = + < 0.1, * < 0.05, ** < 0.01 and *** < 0.001)

This was investigated using a mix of lytic enzymes (Metapolyzyme), avoiding bead beating as it is known to fragment DNA. Metapolyzyme effect was evaluated by qPCR in FFPE blocks fixed for 24 h loaded with *Escherichia*, *Staphylococcus* and *Bifidobacterium* (Fig. 4a (ii))*.* A marked increase in DNA recovery is evident for all three bacterial strains, indicating that Metapolyzyme lyses both G+ and G− FFPE bacteria. The effect of Metapolyzyme was more pronounced in *Staphylococcus*, where treatment increased its recovery by 110× (*p* < 0.001), followed by an 18× (*p* < 0.001) increase for *Bifidobacterium* and a 2.6× (*p* < 0.001) increase for *Escherichia* (Fig. 4a (i)), altogether bringing all strains to the maximum recovery (1 × 10^6^), considering the log decrease of FFPE samples*.* This indicates that a sample lysis step like that exemplified here, must be introduced into the sample prep to guarantee a uniform cross-taxa lysis.

##### Bias introduced by host DNA

To evaluate host DNA-related bias independently from bacterial lysis bias, this was first assessed using paired Protoblocks fixed for 48 h loaded with G− *Escherichia* and *Bacteroides* with (4T1+) or without (4T1−) mammalian cells (Fig. 4b (ii)). Samples were processed maintaining equal ratios of bacterial input. Average DNA concentrations after purification are shown in Fig. 4b. PCR reactions were loaded with 10^6^ cells. As seen in Fig. 4b (i), despite the low 4T1 cell to bacteria ratio, 4T1− reactions had on average a 2× higher bacterial DNA recovery. The same effect was observed later for the G+ *Staphylococcus*. This is not surprising, given the size of mammalian genomes. Eighty-nine percent of 4T1+ total purified DNA corresponded to mammalian DNA. It is likely that with increased host DNA ratios, this effect would become more pronounced.

##### Testing strategies for host DNA depletion

This was tested in Protoblocks loaded with equal ratios of *Escherichia*, *Staphylococcus* and 4T1 cells. Contents of Protoblocks were exposed to known mammalian cell permeabilising solutions (Triton-X, Saponin or Molysis CM solution) and treated with Turbo DNAse [42]. As shown in Fig. 4c, all tested host depletion (HD) strategies significantly reduced the recovery of 4T1 DNA. From these, only Saponin did not significantly affect the recovery of G− *Escherichia*. All treatments tested increased the recovery of G+ *Staphylococcus*, but only Triton-X did so significantly.

#### C. Evaluating DNA damage in terms of fragmentation

##### DNA fragmentation

**Fig. 5:**
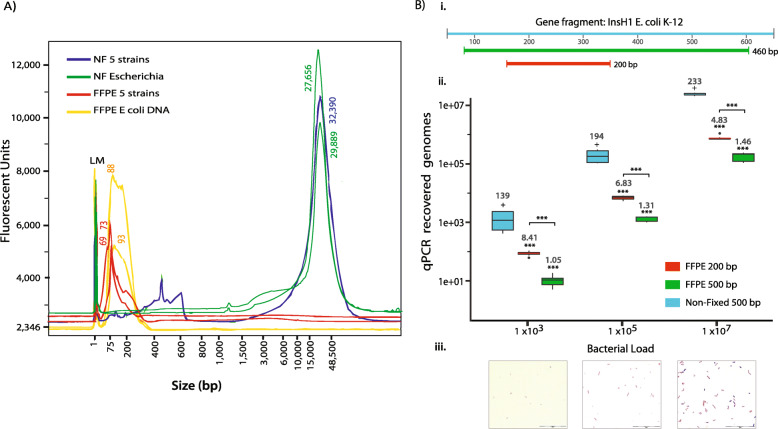
DNA fragmentation in FFPE bacteria. **a** Evaluation of DNA integrity with fragment analyser. Electropherograms of DNA purified from Protoblocks with a mix of 5 bacterial strains (red) and Protoblocks loaded with Escherichia only (yellow) and compared with matched NF bacterial mix (blue) and Escherichia (green). NF bacterial DNA had a higher integrity (GQN > 6.6), while FFPE bacterial DNA from either sample was highly fragmented (GQN ≤ 0.1). No significant difference was observed between Protoblocks or NF samples. GQN = % of DNA above the threshold. The GQN threshold (dotted line) was set to that used for sequencing libraries (10,000). **b** Measuring the recovery of PCR readable DNA from FFPE bacteria in Protoblocks by qPCR. (i) Schematic of primer design for targeted fragments. Both 200 bp and 460 bp DNA fragments target the same *E. coli* K-12 regions. (ii) PCR recovery. Box plot of DNA recovery from 460 bp (green) and 200 bp (orange) FFPE DNA fragments (for each box, *n* = 9) compared with NF DNA (cyan; for each box *n* = 6) normalised to 10^7^, 10^5^ and 10^3^ genomes. Mean recovery of DNA from Protoblocks compared with input DNA significantly differed in both FFPE sample types (*p* < 0.001) as per one-sample Wilcoxon signed-rank test. Fragment length also significantly influenced DNA recovery of FFPE samples (*p* < 0.001), as per Wilcoxon signed-rank test. (iii) Gram-stained slides used for confirming bacterial content. (In all cases, *p* = + > 0.1, * < 0.05, ** < 0.01 and *** < 0.001)

DNA integrity was investigated with a fragment analyser by comparing DNA purified from matched NF and Protoblocks (FFPE) samples containing either a mix of non-fixed (NF) bacteria (ratios as sTable 1) or *Escherichia* only. As seen in Fig. 5a, DNA fragments from NF *Escherichia* (*x̄* = 27,102 bp, %CV = 65.84) or the bacterial mix (*x̄* = 31,100 bp, %CV = 59.19) were highly integral (no fragmentation), with a Genomic Quality Number (GQN) > 6.6, and no significant difference was observed between sample type. On the other hand, DNA fragments from Protoblocks loaded with *Escherichia* (*x̄* = 143 bp, %CV = 41.93) or a bacteria mix (*x̄* = 110 bp, %CV = 53.62) were highly fragmented with a GQN = 0.1 in both sample types. These results were in agreement with FFPE tissue DNA (sFigure 2). These results are comparable with those found in human FFPE samples, where GQN between 0.75 and 2.5 are considered high-quality FFPE DNA and GQN ≤ 0.3 are low and not recommended for sequencing [43].

##### Assessment of PCR readable bacterial FFPE DNA

Since DNA fragmentation of FFPE bacteria was observed to be equal across taxa investigated here (Fig. 5a), the effect of fragmentation on PCR recovery was investigated with Protoblocks loaded with 10^8^, 10^6^ and 10^4^ *Escherichia* cells, as confirmed with Gram staining (Fig. 5a (iii)). Quantitative PCR reactions loaded with 10^7^ (61.2 ± 5.2 ng), 10^5^ (0.8 ± 0.21 ng) or 10^3^ (~ 0.02 ng) bacterial cells were tested for the recovery of a 200-bp (recommended for FFPE) [44, 45] or 460-bp DNA fragment (required for V3-V4 16S rRNA sequencing). This was compared with the recovery of a 460-bp fragment from paired NF (non-fixed) samples (Fig. 5a (i)). While comparable DNA quantities of paired FFPE/NF samples were loaded into the PCR reactions, a significant (> 1-log) reduction was observed in the quantity of DNA recovered from Protoblock samples (*p* < 0.001). A further decline in recovery (3–8×) was evident when targeting longer (460 bp) DNA fragments (Fig. 5a (ii)), a trend that held true across all groups, which varied in terms of quantity of bacteria loaded, thus indicating that DNA fragmentation has a significant effect in the PCR recovery of bacterial DNA (*p* < 0.001).

#### D. Evaluating damage in the sequence quality of bacterial FFPE DNA.

**Fig. 6:**
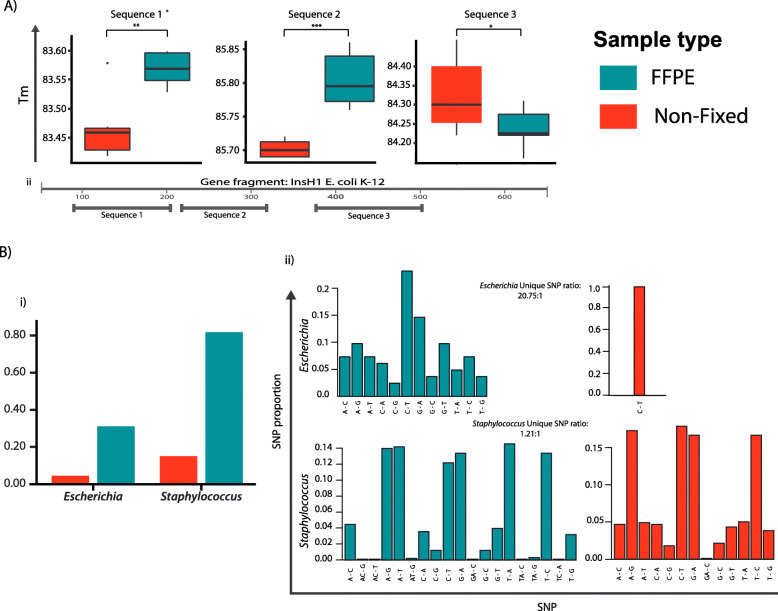
Evaluating sequence quality of bacterial FFPE DNA. **a** Evaluation of DNA sequence aberrations by high-resolution-melt analysis. (i) Box plots of normalised DNA quantities from Protoblock FFPE Escherichia (cyan) and NF Escherichia (orange). Significant shifts in the melting temperatures in 2 of the 3 sequences were observed as per Wilcoxon signed-rank test, with temperature shifts that were on average 0.1–0.5 °C apart from NF counterparts. (ii) Schematic of sequences used for HRM analysis: 3 DNA fragments with an average length of 100 bp were analysed. For each test and each sample type, *n* = 6. **b** Confirmation of sequence alteration by WGS. DNA from Protoblocks loaded with Escherichia and Staphylococcus and their NF paired reference was analysed by whole genome sequencing to determine chimeric reads and single-nucleotide polymorphisms (SNP) against the reference genome *E. coli* K12 MG1655 and *S. aureus* Newman. Here, the SNP are plotted on the *x*-axis and the rate of occurrence on the *y*-axis. Variant calling and level of coverage are measured using SAMTOOLS/BCFTOOLS. (i) Chimeric reads per layer of coverage. (ii) Distribution of SNPs found per bacterial strain

This was assessed in a Protoblock model populated with *E. coli* K-12. Purified DNA was normalised to 10^6^ genome copies. High-resolution melt (HRM) analysis was performed in 3 contiguous DNA fragments (length ≅ 100 bp) that make up a region of the *InsH1* gene (see Fig. 6a (ii)). To determine the presence of any sequence aberrations in Protoblock FFPE DNA, their melting temperature (Tm) was compared with that of NF DNA and the differences measured. Figure 6a (ii) shows the final Tm for each fragment investigated. Tm shifts with variable levels of significance were observed in all fragments. This is indicative of a change in the underlying DNA sequence, as would be expected in a clinical FFPE sample. To confirm these results, DNA purified from Protoblocks loaded with *Escherichia* and *Staphylococcus* and their paired NF samples were analysed by WGS. Findings from the DNA melting temperature analysis correlated with the results of WGS. For both bacterial strains, a higher number of sequence artefacts (chimeras and SNPs) were found in FFPE samples, when compared with their NF reference (see Fig. 6b).

#### E. Characterising common contaminants in the FFPE and 16S rRNA gene amplicon sequencing workflow

**Fig. 7:**
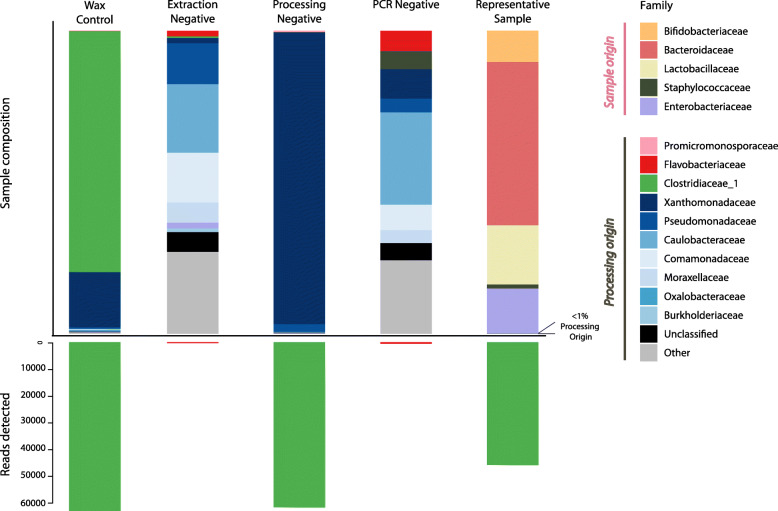
Evaluation of sources of environmental contamination and their effect on Protoblock samples. Composition bar plot per sample showing proportional composition of bacterial taxa per negative control, with corresponding number of reads detected by 16S rRNA gene sequencing. Compared with representative Protoblock sample

The Protoblock is susceptible to contamination in a similar way to clinical FFPE samples. The priority of the fixing process is to preserve the tissue for later histological analysis, not to prepare a sample suitable for a high-throughput bacterial sequencing. In this instance, contamination was detected as shown by the number of reads in the negative controls (Fig. 7). It is unlikely to have had a significant effect on the overall biological signal in this instance, given that the bacterial reads detected, and their taxonomic classifications, differ completely from those of the Protoblocks analysed. It must be stated that the level of bacterial biomass loaded into the Protoblocks is orders of magnitude higher than what can be expected in a clinical FFPE sample, and as such the level of contamination present in the negative control samples poses a significant risk to the accuracy of any sequence-based analysis of clinically collected FFPE samples,

## Discussion

FFPE tissue specimens are an invaluable resource that has driven research in human cancer genomics, where numerous workflows have been developed for these samples. Over a decade of study has revealed that FFPE DNA damage is influenced by many factors during processing and storage. This results in a high inter-sample variability in the degree of DNA damage, with some samples being unsuitable for sequencing analysis [46]. To address this, the development of a robust quality control (QC) system has been crucial in directing workflows maximising the recovery, while guaranteeing the fidelity of analysis outputs. Most notable among these are the analysis of DNA fragment length (fragment analyser) and PCR readability of DNA in a sample (Infinium FFPE QC, Illumina).

Likewise, before any reliable and reproducible use of FFPE samples for microbiome analysis can be performed, a robust QC system must be developed and systematically implemented. The Protoblock presented here represents a highly relevant starting point. This method is advantageous in that the cell populations and fixation strategies can be adapted to meet the requirements for sample type and sample prep/sequencing workflow to inform on their effects on analysis outputs [47]. Ideally, a mildly fixed version of the Protoblock (bacteria and cells embedded in agar) would be developed in a standardised manner in specialist facilities. These could be used prospectively by researchers/clinicians so that FFPE processing is in line with the methods applied in their hospital/clinic/lab and performed at the same time tissue samples are processed. However, this method could also be adapted by researchers with specialised needs. In this case, commercial standards compatible with the Protoblock or FFPE workflow would highly facilitate the in-house development and at the same time guarantee sample accuracy and reproducibility.

It has been shown here that the Protoblock is a representative FFPE model, since its contents are exposed to the same processing as FFPE experimental samples and has the same degree of DNA damage (fragmentation, PCR recovery and sequence alteration) as clinical FFPE tissue samples. Moreover, the degree of DNA damage in the Protoblocks can be modulated by changing the fixation time. This advantage can be exploited to develop a system similar to Infinium FFPE QC (Illumina), where a sample with a good DNA quality score serves as a standard and Cq deviations from this inform on the suitability of samples for sequencing analysis. The Protoblock can also serve as a quantitative standard to determine cycle number at which tested FFPE samples will have detectable levels of 16S rRNA gene sequences, if any.

The Protoblock was also proven effective as a control for optimising the sample prep workflow for microbiome analysis. Here, it informed on expected sources of bias, such as that introduced by host DNA, while at the same time allowing for the assessment of host DNA depletion strategies. The Protoblock also educated on bias introduced by the bacterial lysis strategy included in the sample prep used (an impact that has been well documented) [48, 49]. From the results shown here, it is clear the QIAGEN FFPE DNA sample prep is unsuitable for microbiome analysis, since it is strongly biased towards Gram-negative bacteria. However, due to the lack of a standardised sample prep methods for microbiome analysis specifically designed for FFPE tissue specimens, this sample prep method has been used in several microbiome studies to date. Therefore, the microbiome research field is in dire need of standardised procedures for the processing of these samples.

This study provides foundational evidence of treatments that can aid the development of such methods: (1) For bacterial lysis, treatment with bacteriolytic enzymes, such as Metapolyzyme, can improve the cross-taxa representation while not imposing risks for DNA fragmentation that bead beating would. (2) The large fraction of host DNA background could be reduced using mammalian cell–selective permeabilisation agents compatible with fixed dead cells. Here, Saponin proved effective when used in combination with a DNAse. (3) Incorporating procedures that alleviate the severity of DNA template degradation can improve DNA fragment length and sequencing quality. A sample prep method that incorporates such treatments can render a higher quality of DNA, more compatible for sequencing studies, and results that are less influenced by bias, thus improving the reliability of the results.

An unexpected finding was a higher than expected recovery of FFPE *Bifidobacterium* in samples processed without undergoing bacterial lysis. The opposite was found for *Staphylococcus.* This persisted across all experiments and in multiple Protoblock batches (*n* > 30). This reinforces the need to thoroughly study the effect of FFPE on bacteria prior to any microbiome analysis of FFPE specimens. Principally, a thorough investigation on the effect of FFPE in bacterial membrane/cell walls should be conducted, and also the bacterial DNA itself.

Finally, contamination is a considerable threat to the accuracy of sequence-based analysis of low biomass samples such as FFPE specimens. Steps in the processing of FFPE samples require the use of solutions that are difficult to keep sterile, and contamination from these sources could easily obscure the true results in cases of low microbial load. Use of a contamination control system, such as the that proposed here: combination of an empty (agar only) and a bacteria-loaded Protoblock along with a sample of the paraffin wax used for embedding, can inform on the most common contaminants and the level of contamination introduced by any processing of FFPE samples required, in advance of a sequencing study to assess their potential influence on the studies and address their bioinformatic or biological removal. As mentioned earlier, the Protoblock contained both a known bacterial community and a comparatively high level of bacterial biomass, and therefore, the effect of contamination was negligible. However, as neither of these features are typical of clinical FFPE samples, researchers should be mindful of the susceptibility of these samples to environmental contamination.

Given the wide availability of FFPE specimens, these represent a huge potential as source material for microbiome research, especially for rare or difficult to attain samples. However, results shown in this study clearly indicate that performing microbiome studies on FFPE material has severe limitations that should not be taken lightly. For these samples to become accessible for microbiome research, dedicated workflows for this sample type must be developed and optimised. These must include sample prep methods, quality control system and a contamination control system. Only after such systems are in place can the microbiome field consider the reliable, reproducible and accurate microbiome analysis of the of FFPE specimens.

## Conclusion

FFPE tissue is still far from ideal for microbiome studies. However, given the limited availability of rare ‘fresh’ samples, unlocking the potential of FFPE samples for microbiome analysis could have a huge effect on the field. For this to be a reality, a robust quality control system, including standards, needs to be developed. While FFPE microbiome research is still in dire need of optimisation, the Protoblock is well placed for use in optimisation of methods in order to move the field forward.

## Methods

### Preparation of Protoblocks

#### Moulds

Moulds used to make cylinder-shaped disks were made from a 54 × 11 mm adapter tube with a flat base (SARSTEDT, Cat No. 55.1570).

#### Cell culture

*Mus musculus* mammary gland cancer cells (4T1) were grown at 37 °C 5% CO_2_, in RPMI-1640 (Sigma-Aldrich) media supplemented with 10% FBS (Sigma-Aldrich), 100 U/mL penicillin and 100 μg/mL of streptomycin (ThermoFisher), and counted with a NucleoCounter® NC-100™ (ChemoMetect, Copenhagen).

#### Bacterial growth conditions

*E. coli* K12 MG1655 or *E. coli* Nissle 1917 carrying a P16Lux plasmid [50] were grown aerobically at 37 °C in Luria-Bertani (LB) medium with 300 μg/ml Erythromycin (Sigma-Aldrich). *Staphylococcus aureus* Newman (ATCC 25904) was grown aerobically at 37 °C in Todd-Hewitt broth (Sigma-Aldrich). *Bifidobacterium longum 35624* was grown anaerobically at 37 °C for 24 h in MRS medium (Sigma-Aldrich). *Lactobacillus amylophilus* (ATCC® 49845™) was grown in MRS medium (Sigma-Aldrich) at 30 °C in 5 % CO2 for 24 h. *Bacteroides thetaiotaomicron* (ATCC®29741™) was grown anaerobically at 37 °C for 24 h in FAB medium (NEOGEN, Lancashire, UK). Bacterial cultures were harvested by centrifugation and suspended in PBS. A 1 ml aliquot of the suspension was used for to count colony-forming units (CFU) by retrospective plating. The rest was resuspended in Neutral Buffered Formalin and left to fix for 18 h at room temperature (RT).

#### Counting fixed bacterial cells

The cell suspension was counted using a bacterial counting kit for flow cytometry (Invitrogen). In brief, a 10% aliquot from the bacterial suspension was serially diluted to 1 × 10^6^ cells in 989 μl of NaCl. Bacterial cells were stained with 1 μl of SytoBC, and 10 μl (1 × 10^6^) of counting beads was added to the suspension. Cells were counted in an LSR II Flow Cytometer (BD Biosciences). The acquisition trigger was set to side scatter and regulated for each bacterial strain to filter out electronic noise without missing bacterial cells. This value was approximately 800. The volume corresponding to approximately 2 × 10^7^ CFU of each bacterial strain and 2.2 × 10^7^ 4T1 cells were mixed together.

#### Fixing cells in an agar matrix

An equal volume (270 μl) of sterile agar (1.5X of elution specified by the manufacturers) pre-aliquoted and kept at 56 °C was pipetted into the cell suspension and thoroughly mixed by vortexing. The mixture was pipetted into the moulds and left to solidify for 3 min at RT. Once solidified, the disk was placed in 5 ml of formalin for an extra 24 h for 48-h fixation blocks or immediately processed for 24-h fixation blocks.

#### Dehydration and paraffin embedding of cell disk

Cell disks were placed into a processing cassette and processed automatically with a LOGOS J (Milestone Medical, Bergamo). Here, they were dehydrated for 4 h with increasing concentrations of ethanol (37 °C), cleared 2X with xylene for 2 h 20 min and 2X with isopropanol for 1 h 40 min at 37 °C, and 1X with isopropanol for 50 min at 60 °C. Finally, the blocks were embedded in paraffin for 8 h 32 min at 62 °C. Once paraffinised, the Protoblocks’ volume, diameter and height were measured with a calliper and by volume displacement [51]. Processed Protoblocks were placed in a 1.5 × 1.5 cm embedding mould and mounted to a processing cassette. All Protoblocks used in this study were stored for no longer than 12 months prior their analysis.

### Confirmation of cell content by microscopy

#### Sectioning

The blocks were sectioned using aseptic technique, either at 4 μm for imaging or at 10–20 μm for DNA purification. The cell load of each slide was calculated by multiplying the total bacterial load by the volume of each slide.

#### Immunofluorescence and histochemistry

Cell integrity was evaluated with Gram staining (Sigma-Aldrich) or H&E staining with Mayer’s haematoxylin (Sigma-Aldrich). Bacterial counts were confirmed in 3 sections stained with either 1:50 α-*Escherichia coli* (Abcam, 137967) or 1:400 α-*Staphylococcus aureus* (Abcam, 20920), counterstained with either Alexa Fluor 488 (Jackson Immunoresearch Laboratories Inc., USA) or Alexa Fluor 555-conjugated (Abcam 150062) donkey anti-rabbit Ig. Stained sections were mounted in ProLong Gold antifade reagent with DAPI (Invitrogen, UK). Gram-stained sections were counted in a bright field using an Olympus BX51 microscope, with a × 100 lens. Immunofluorescent stained slides were counted at × 20 (4T1 cells) or × 60 (bacteria) with a fluorescence microscope (Evos FL Auto). For each slide, at least 20 randomly selected fields of view were counted. The area of the field of view (FOV) was recorded using the microscope’s software and used to calculate the volume counted.

### DNA analysis

#### DNA Purification

For purifying DNA from Protoblocks, unless specified, 10 × 15 μm sections aseptically collected were deparaffinised with 2X xylene washes and processed following procedures specified in the QIAGEN FFPE DNA kit protocol (Qiagen Inc., Valencia, CA, USA). This kit does not include bead beating. Therefore, bead beating was not performed. DNA was eluted in Tris-HCL buffer and quantified with a Qubit™ dsDNA HS Assay Kit (Invitrogen, USA). For non-fixed bacteria, bacterial cultures were grown to an OD_600_ of 1. Two-milliliter aliquots were processed following procedures of the GenElute™ Bacterial Genomic DNA Kit Protocol with Lysozyme and Lysostaphin (Sigma) (without bead beating) and eluted in 50 μl of Tris-HCl. In all cases, DNA was stored at − 20 °C until further analysis.

#### Fragment analysis

One microliter of DNA purified from FFPE blocks was analysed in an Agilent 21000 bioanalyser using a High Sensitivity DNA kit (Agilent, Cat. No. 5067-4626). For Genomic Quality Number (GQN), the threshold was set to 10,000 bp and the ratio of DNA above this threshold measured for each sample. Average fragment lengths and %CV from area underneath a maximum peak were also measured.

#### Quantitative PCR (qPCR)

For dye-based qPCR, reactions were prepared using LUNA Universal qPCR master mix (NEB, USA) and 0.25 μM of each primer (sTable 2). Multiplex qPCR reactions were prepared using LUNA Universal Probe qPCR master mix (NEB, USA) and 0.5 μM of each primer (sTable 2) and 0.25 μM of probe for each strain. Reactions for simultaneously quantifying three bacterial strains were set using the fluorochromes: FAM, HEX and CY3. The thermal profile included a 1 min at 95 °C initial denaturation, followed by 40 cycles of denaturation at 95 °C × 10 s, annealing for 15 s at the temperature specified by NEB’s Ta calculator for Hot Start Taq, followed by 20–40 s of extension at 68 °C. For each assay, a 5-point standard curve was made from log_10_ dilutions of a gene block corresponding to species-specific genetic regions, using an initial concentration of 10^6^ copies. Primers and gene-blocks were acquired from IDT (Coralville, USA) (see sTable 2 and sMaterial 1). Efficiency between 95 and 105% and R-square values > 0.995 were deemed as acceptable. All samples were run in triplicate.

#### qPCR melt curve analysis

For melt curve analysis, FFPE *E. coli* DNA was normalised to 1 × 10^6^ copies/μl. Reactions were prepared using 1X NEB Luna probe qPCR mix, 1.25 μM EvaGreen Dye (Biotium, CA, USA), 37.5 nM ROX as a reference dye, 0.25 μM of each primer (sTable 2) and 2.5 μl of template DNA. Cycling conditions used are as described for absolute quantitation with addition of a final extension step of 2 min at 68 °C. This was followed by high-resolution melt analysis set to read fluorescence every 0.2 °C with 10 s soak time from 65 to 95 °C. Values for the first derivative of the normalised fluorescence multiplied by − 1 were exported and analysed in R environment, v3.4.4.

#### 16S rRNA sequencing library preparation

Amplification of the hypervariable V3–V4 region of the 16S rRNA gene (see sTable 2) was performed in 50 μl reactions, containing 1X NEBNext High Fidelity 2X PCR Master Mix (NEB, USA), 0.5 μM of each primer, 8 μl template (5-15 ng/μl) and 12 μl nuclease-free water. The thermal profile included an initial 98 °C × 30 s denaturation, followed by 25 cycles of denaturation at 98 °C × 10 s, annealing at 55 °C × 30 s and extension at 72 °C × 30 s and a final extension at 72 °C × 5 min. Amplification was confirmed by running 5 μl of PCR product on a 2% agarose gel. Hereafter, procedures were performed as per the Illumina 16S Metagenomic Sequencing Protocol (Illumina, CA, USA). PCR products were cleaned, and sequencing libraries were prepared using the Nextera XT Index Kit (Illumina). Libraries were cleaned and quantified using a Qubit fluorometer (Invitrogen) using the ‘High Sensitivity’ assay. Further processing was performed by GENEWIZ (Leipzig, Germany) where samples underwent a 300-bp paired-end run on the Illumina MiSeq platform.

#### Negative controls

(i) Processing control: sterile agar was exposed to the complete FFPE processing workflow. (ii) Wax control: wax was taken from the edges of an FFPE block. (iii) Sample prep control: this was included by running an empty sample prep reaction. (iv) PCR control: a 16S PCR reaction was loaded with microbial DNA free water.

#### WGS sequencing library preparation

For NF controls, DNA from bacterial cultures of *Escherichia coli* MG1655 and *S. aureus* Newman were grown as per the ‘Bacterial growth conditions’ section to and OD_600_ of 1, and their genomic DNA was purified using the GenElute™ Bacterial Genomic DNA Kit Protocol with Lysozyme and Lysostaphin (Sigma). For FFPE bacteria, DNA from Protoblocks containing either strain was purified using the QIAGEN FFPE kit. In all cases, DNA was eluted in 50 μl of Tris-HCl. Total purified DNA was sent to GENEWIZ (Leipzig, Germany) where WGS was performed using 2 × 150 bp chemistry on an Illumina HiSeq.

### Murine models

#### Animals, mammalian cell culture and tumour induction

Murine experiments were approved by the Health Products Regulatory Authority (Dublin, Ireland) and the Animal Experimentation Ethics Committee of University College Cork (Cork, Ireland). RENCA cells were grown in RPMI media (Sigma) + 10% FBS (Sigma) and counted with a NucleoCounter (ChemoMetec). Tumours were induced in 8-week-old BALB/c mice by subcutaneous injection of 1 × 10^6^ cells suspended in 200 μl serum-free RPMI media. Tumours were measured daily with a Vernier calliper, and their volume was calculated by measuring their longest diameter, and at the diameter perpendicular to this.

#### Bacterial preparation and administration

Bacteria were prepared for administration once murine tumours were approximately 5 × 5 mm in diameter. *E. coli* Nissle 1917 was grown to an OD_600_ of 0.8 in LB media, with 300 μg/ml erythromycin, harvested by centrifugation and washed 3X with PBS. *Bifidobacterium breve* UCC2003 was grown anaerobically for 24 h in Man, Rogosa and Sharpe (MRS) media (Oxoid), + 0.05% l-cysteine hydrochloride (Sigma), and harvested and washed 3X with PBS + 0.05% l-cysteine. Both bacterial strains were serially diluted to 1 × 10^7^ CFU/ml. Tumour-bearing mice were administered 100 μl of either bacterial suspension or PBS (negative control) via lateral tail vein injection, as per [50]. Bacterial counts were confirmed by retrospectively plating in LB agar supplemented with 300 μg/ml erythromycin (*E. coli*) or RCA supplemented with 50 mg/L mupirocin (*B. breve*).

#### Bacterial recovery from mice

Mice were culled 7–11 days after bacterial administration. Tumours were aseptically excised and halved. One half was placed in 10% buffered formalin and fixed for 24 h at RT. The other half was placed in 1 ml PBS (+ 0.05% l-cysteine for *B. breve*) and homogenised using a 70-μm nylon cell strainer (Corning). Cell strainers were washed with 1 ml PBS. Homogenised tumours were serially diluted with PBS and plated for retrospective counting as per [52].

#### Formalin-fixed tissue processing

Formalin-fixed murine tissues were placed between two biopsy pads (Kaltek) in a histology cassette and processed using a LOGOS J Hybrid Tissue Processor (Milestone) and paraffin embedded as per the ‘Dehydration and paraffin embedding of cell disk’ section.

#### DNA extraction and analysis of FFPE tissue

The 8 × 10 μm sections were processed for each specimen. Samples were subsequently processed with a QIAamp DNA FFPE Tissue kit (Qiagen) per the standard protocol, with the following exceptions: Tissue was deparaffinised with 2X xylene washes and the incubation with Buffer ATL and Proteinase K was performed for 1 h 45 min. DNA was eluted in 35 μl Buffer ATE. Quantitative PCR reactions were set up as per the ‘Quantitative PCR (qPCR)’ section, using primers and probes specified in sTable 2.

### Bioinformatics and statistical analysis

#### Statistical analysis

All statistical analyses were performed in the R environment, v3.4.4, using methods stated in the figure legends.

#### 16S rRNA gene sequence analysis

The quality of the paired-end sequence data was initially visualised using FastQC v0.11.6, and then filtered and trimmed using Trimmomatic v0.36 to ensure a minimum average quality of 25. The remaining high-quality reads were then imported into the R environment v3.4.4 for analysis with the DADA2 package v1.8.0. After further quality filtering, error correction and chimera removal, the raw reads generated by the sequencing process were refined into a table of Amplicon Sequence Variants (ASVs) and their distribution among the samples. As the aim was to characterise if contamination is present, rather than to remove it, negative controls were included to compare with the FFPE Protoblocks, with no further action taken. Bacterial sequence reads were classified using the Mothur classifier [53], trained on the RDP database (v11.4).

#### Variant calling from whole genome sequence data

*Filtering*: HiSeq sequence data was quality filtered. Only very high-quality bases were considered, to minimise the risk of sequencing errors causing false positive variants. Short fragments were also removed to reduce the likelihood of spurious alignments of regions from contaminant bacterial genomes. Trimmomatic was used to remove all reads shorter than 50 bp in length and to trim reads when the average per base quality in a sliding window of size 4 dropped below 30.

*Alignment*: Of the three possible Burrows-Wheeler alignment tools, the BWA-mem aligner was used as the average read length was 150 bp, and BWA-mem is recommended when reads are over 70 bp in length as per the manual reference pages [54]. Default settings were used with the exception of allowing alignments with a minimum score of 0, rather than the default 30 as we were unsure of the extent of DNA damage–induced sequence alterations. Given the stringent parameters used for read length and quality filtering, relaxing the minimum alignment score gave the best possible chance of variant detection. Samples were aligned to the original reference genome, *E. coli* MG1655.

*Variant calling*: Variant calling was done with BCF tools, using the BCF call function. The variants were then filtered using the norm and filter functions within BCF tools. Filtering was performed to remove variants when the read depth was below 10, the quality was below 40, or when the variant identified was not supported by both the forward and reverse read of a read pair.

## Supplementary information

**Additional file 1.**

## Data Availability

Both 16S and WGS sequence data will be made available on the SRA upon acceptance of the manuscript. Additional data sets and material is available upon reviewers’ request.

## Abbreviations

BSA: Bovine serum albumin CFU Colony-forming units FFPE Formalin-fixed paraffin-embedded H&E Haematoxylin & eosin HRM High-resolution melt analysis LB Luria-Bertani NF Non-fixed NGS Next-generation sequencing PBS Phosphate buffer saline PCR Polymerase chain reaction qPCR Real-time PCR RT Room temperature Tm Melting temperature WGS Whole genome sequencing
