# Supplementary material for: Protoblock - A biological standard for formalin fixed samples
Source: Microbiome. 2020 Aug 22;8:122. doi: 10.1186/s40168-020-00901-1 (PMC7443293; doi:10.1186/s40168-020-00901-1)
Supplement: Supplementary file 2 — Additional file 1. [file 40168_2020_901_MOESM1_ESM.docx]

# SUPPLEMENTARY MATERIAL

***
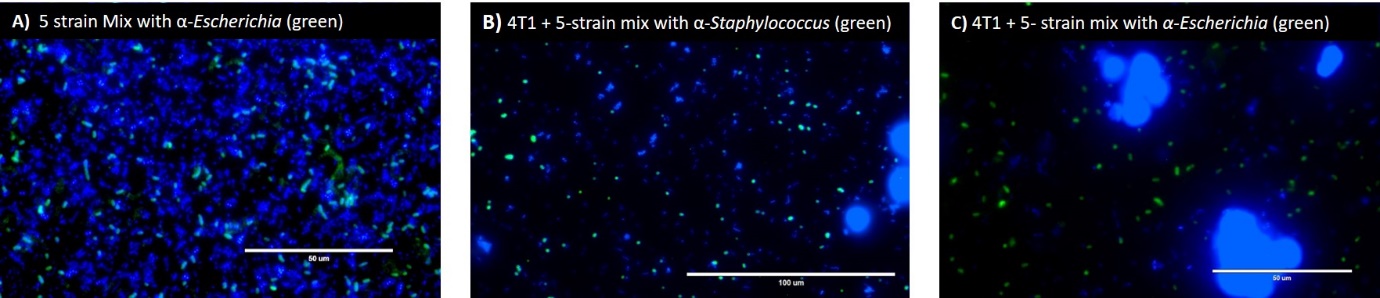
***

***Supplementary Figure 1. Microscope images of Protoblocks with 5-strain mix contents***

***A)*** *Microscope* *image (40X) of Protoblock loaded with the 5 bacterial taxa specified in Figure 2. DAPI (Blue), staining all bacterial cells. In green, α-Escherichia.*

***B)*** *Microscope* *image (40X) of Protoblock loaded with the 4T1 cells and the 5 bacterial taxa specified in Figure 2. DAPI (Blue), staining 4T1 and bacterial cells. In green, α-Staphylococcus.*

***B)*** *Microscope* *image (40X) of Protoblock loaded with the 4T1 cells and the 5 bacterial taxa specified in Figure 2. DAPI (Blue), staining 4T1 and bacterial cells. In green, α-Escherichia.*


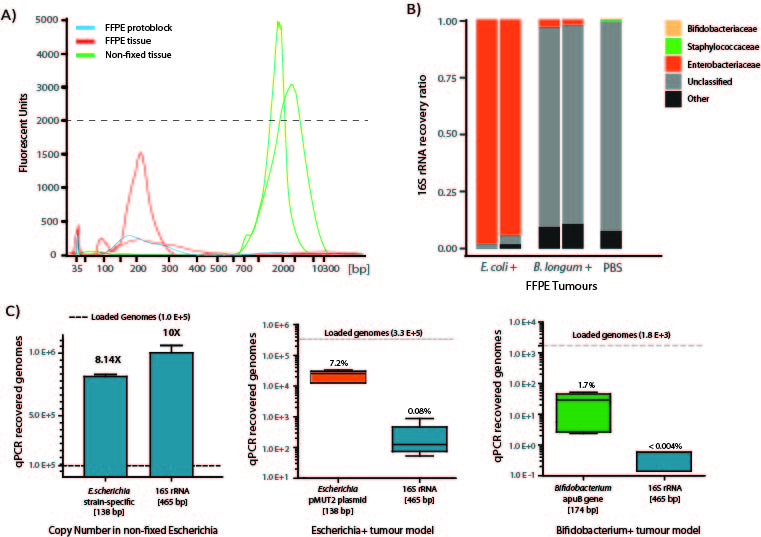


***Supplementary Figure 2. Validation of findings in FFPE tissue.***

***A) DNA fragmentation.*** *Electropherograms comparing the integrity of NF tissue DNA (green), with FFPE tissue DNA (red) and the contents of Protoblocks (blue). NF tissue fragment length = 4,406 ± 1,939 bp, DNA from FFPE tissue = 229 ± 20 bp and DNA from Protoblocks = 192 ± 48.5 bp.*

***B)*** ***16S rRNA recovery of FFPE tissue.*** *Bar plot* *showing bacteria recovered by 16S rRNA sequencing from murine tumours models loaded with either Escherichia, Bifidobacterium or PBS. Here, Escherichia was readily detected, while Bifidobacterium was not detected.*

***C) PCR recovery of Escherichia and Bifidobacterium from FFPE tissue. i) Assessment of strain specific gene and 16S rRNA gene in the recovery of non-fixed E. coli.*** *Bar plot showing the number of gene copies retrieved for either a strain specific gene ( = 8.14 ± 0.43 copies) or 16S rRNA gene ( = 10 ± 1.2 copies) after amplifying an input of 1 x 10^5^ Escherichia cells.* ***ii) Recovery of Escherichia from FFPE tumours.*** *Box plot showing the PCR recovery of an input of 3.3 x 10^5^ Escherichia genomes with a strain specific 137 bp DNA fragment (2.33 x 10^4^ ± 8.8 x 10^4^ genomes) and that of the 16SrRNA gene (2.7 x 10^2^ ± 3.2 x 10^2^ genomes).* ***iii) Recovery of Bifidobacterium from FFPE tumours.*** *Box plot showing the PCR recovery of an input of 1.8 x 10^3^ Bifidobacterium genomes with a strain specific 174 bp DNA fragment (3.2 x 10^1^ ± 21 x 10^1^ genomes) and that of the 16SrRNA gene for which there was not a reliable amplification detected (only 2/6 replicates returned an average amplification of 7 x 10^-2^  copies.*

**Geneblock Sequences**

***Bacteroides thetaiotaomicron* (unknown gene)**

**780 bp**

**Accession No. AF182955.1**

Accattcgggttggagttttactttgaatggacaatctagcgacttgtatgattatgttccggatggcagcatacttgatatcgatttgggaaaacaagagaatataaaaactattgctttacatttctatgaatggttttattcttcggaaagtgcaagtatagcgataagtaatgatggagagaaatatgaggatcttggtgtagcttcaggatttgcaaataagacaagttacattcttcttcttgtggctaaacaagcgcaatatataagagtaactttccatggagctctttattattctccttatattaatactgtaggtatttatactgaaactgaataaaaaatggcccaagtttattactgaatgagcttgggctatttgctgtttatatcatggtgtgatcagttctaagttttctttgttaataaaacttagaactgatttttttcttgagtatcttatatttttggattgtattatctaaaaactaattaaaggaaatgctatgagaatcattcgccgtattatttttcagctgctttgtttgttgggagcatgttgttatattcctgcaacagcacaagtcgttttagttgataatggaaaaactaaatccaggattattctatcagaaaatgaccagattaatcaaatatcagcaaatttatttcaattgttccttcagagaatttcaggttgtacatttcccattgtaaaagggcagaatgcaaaaaaaggagatattataatcagtagcaaaaccccagctaca

***E. coli* k-12 MG1655 (InsH1 Transposase NC_000913.3)**

**Accession = NC_000913.3:274193-274818**

**626 bp**

GCAACCCCTTGTATCTGGCTTTCACGAAGCCGAACTGTCGCTTGATGATGCGAAATGGGTGCTCCACCCTGGCCCGGATGCTGGCTTTCATGTATTCGATGTTGATGGCCGTTTTGTTCTTGCGTGGATGCTGTTTCAAGGTTCTTACCTTGCCGGGGCGCTCGGCGATCAGCCAGTCCACATCCACCTCGGCCAGCTCCTCGCGCTGTGGCGCCCCTTGGTAGCCGGCATCGGCTGAGACAAATTGCTCCTCTCCATGCAGCAGATTACCCAGCTGATTGAGGTCATGCTCGTTGGCCGCGGTGGTGACCAGGCTGTGGGTCAGGCCACTCTTGGCATCGACACCAATGTGGGCCTTCATGCCAAAGTGCCACTGATTGCCTTTCTTGGTCTGATGCATCTCCGGATCGCGTTGCTGCTCTTTGTTCTTGGTCGAGCTGGGTGCCTCAATGATGGTGGCATCGACCAAGGTGCCTTGAGTCATCATGACGCCTGCTTCGGCCAGCCAGCGATTGATGGTCTTGAACAATTGGCGGGCCAGTTGATGCTGCTCCAGCAGGTGGCGGAAATTCATGATGGTGGTGCGGTCCGGCAAGGCGCTATCCAGGGATAACCGGGCAAACAGA

***S. aureus* Newman (Thermonuclease gene)**

**Accession = CP023391.1**

**766 bp**

ATGAAGTCAAATAAATCGCTTGCTATGATTGTGGTAGCCATCATTATTGTAGGTGTATTAGCATTTCAATTTATGAATCATACGGGTCCTTTCAAAAAGGGGACGAATCATGAACCTGTACAAGATCTAAGTGGTACAGATAGAGTACATGTTCAACGAGTTGTGGATGGTGATACATTCGTTGCAAATCAAGATGGTAAAGAAATCCAAGTTAGGCTTATAGGGGTTGATACGCCAGAAACGGTGAAACCGAATACGCCTGTACAACCATTTGGCAAAGAAGCATCAACGTATAGTAAGAAGACATCCACAACTCAAGATGTTTAGTTAGAATATGATCAAGCCAAACAAGATCGCTATGGTAGAACATTGGCGTATGTATGGATAAGTAAAGATCGTATGTACAATAAGGAATTAGTGGAAAAGGGACTTGCTAGAGAGAAGTATTCCTCACCAGATGGCACACGTAGACCTGTATATATAGAAGCACACGATACAGCTAAACAACAGCAATTACATATCTGGAGTAACGAATTAAATAGGTGAGCATATACCTTAGAGTAACGTATACTTGAAGCATCGATTTATTATTACCATAACTCCATACGCAAAGAAGTAATTTAAAGGCATAATAACCATAGTGCGTATCGAGCGTAATGGATTATTATGCCTTTGATTTTACTTGAATATAGCTGTGATTTGATCAACCTGTTCTTTATTTAAAGGGTGTTTAGATAGCTCTATGGCTTCGCTAATAATACGTCGT

***Lactobacillus amylophilus* (rpoA gene)**

**Accession = HE573908**

ttacggcagattcgtaattgaacccttggaacgtggttatggtactactttaggaaattcacttcgtcgtgttttactagcttctttaccaggaagcgcagttagttatctccaaattgatggtgttttacatgaattttcaacaattcctggtgtattagaagacgttacacagattattttgaatcttaagaagcttaccttaaagtcaatcgcggctgatgaaaagttagcagaaatcgatgtagaaggtcccgcaactgttactgccggcgatcttaaagttgatgatgaagttcaaattttgaaccctgatcagtacatcgctacggttgctgagggagcacatcttcggatgactgttgccattaaacagggtcgtgggtatgttccagcggaccagaataaatccgatgatatgccgattggtgttgtgccagttgattcactattttcacctatcgaaaaggttaactaccaagtcgaaggaacccgtgttggacggaacaacgactatgataagttaacgatggaagtttggacggatggctctattacacctaacgatgcattgagttttggtgccaagattttaaccgaacacctaaacgtgtttatgaatacaaatcttgtaactgagttcactgatgtgatggtggagaaggaagatactaagaaagagaaacaa

***B. longum* 35624**

**Accession No. CP013673.1:461108-461924**

**817 bp**

Gatatccaaattatgtgccgtaatgggtatgcggaaccgccgaatgcgcgtcgtcgtctgattcgtaagcatgaaacactgcttaagtatttcgagaaacgatatggagatttcgcttctggctatcgctataaggtaaatcttccagccgtaccggaggaactcaaaggttgcatctggttatgctggtggcaagggcttgaccatgctccgcgtatcgtccaacgctgtgtagaatcgattcgcgaatatgcgggagatcgacgtgtgattgtgattaccgaggacaattatcgtgattatgtgaccttccctgactggatgatgcgcaaatatcatgaaggcactattgctcgcacacatttgtcggacttgctgcgtttatcgttgctggctcagtatggcggattgtggttggacgcgacgttctattgcgctggcgatattgaagactgttttggccagccattgttctctatcaagcgccccgagtatttgcatgcaagtgtggcgcagggatattttgcaaattactcttttggatgtgatgccgaccatcgttggatatttgccgtgatacgtgatttcttgcttgaatattggcgtacaaatgattttatggtcgactacttgttcttggattatttaatcgtactagtgcaacgacattgcactgcggttaaggaggcgtttgcagcaatcacccccaataatccacaatgcgatgacctgttcaagtgtttaggtgatgtctttaatgagcgtcattgggagtcattaattgccgatacatcattgttcaaattgacatggaagc

***E. coli* Nissle (plasmid pMUT2)**

**Accession No. CP023342.1**

**1173 bp**

tcaccgaggccttgaaattaagctcagggaacatacagaccgctatccaagaagagaacgacagccagctaaaacagatacaccgcttagtcggaatgacatggctgtacagcctggcattaagtgggatcctgtttgcgatattgattggagtagcttggtatctcgggactatcgtggtcgaacgccagaacgaaatcagcgagcagagccagatcctgcaggacttaaagagccagaccggagccggcgtatcgataattcacgattccaagaacaagagcgtgtattacctgatccttccgcagggggcgaagaagatcgacgagtacaagaacgctcaacatcgtcaggtcatcaagtacagcgccaaataacctcatcagacgccacagaatcgattctgggcggttttatctatcagggtgaagagattcatgaccgaaatggagcagcagcttctgagcgcattagagagcttacagaggcactacgaacaacagcagcaagcgtggcaggacagctacgccaacttacagcgcatgttcgaggttacctcgcaggagttggcgaaaaacgacagggtttgtcaggccttgagcatgcaagtcaccggcttggcgcagcaagtcgagagcttaaacagaacagtgcgccgcttgagcaattagccaagcggcacgaacagcggtattctcgcggtcacggcatgagtttataagcgtttatcggccgcatcgtaagcggcagaacgctcgcgcttaccgaccgccaccacgaataccgtaatggtttgatcgcgaacctgatagaccaagcgataaccggatgcacggagcttgattttgtagcagtcatgcagctctcgcaggcgatttttatcgatccgcgggtgttgtagaacctgctcgagttttttcttgaactgcagacggacatcatccccgagcttgcgccattccttcagggctcggggatcaaattcaaggttatagctcatccagtgacacctttacgcccgcctgtgggttttccagacgatcccgaacgatagccatcaaatcggcatcatcctcggtcagcaaaacctgctggaacggcaaacgtccgctttgggccacatattccagtgtttggcgcagaacctcggacggcgttacgcccagcttttcc

***B. breve* UCC2003**

450 bp

tggatggatgggtcccagatatggaaatgcgagtcgatgatcctctgcatgtcgcgtgtcctagtcgagtgccctgtcgaggtggacgtatccgccgtcggggaagacccactgtccggtggtgtgcgaggacctgtcggacagaaggaaaaccacggtgtccgcgatttccgaagtctcggtcatacggtgcccaaaaggaatcctgtcggtgatcttcgccaactgctcgcgctgcgcctgctcgtcgccgaacgtcttgatccattgcgcgtacagcggtgtccatgcttcggccacgacgacggcattgacccggacctcatccggggcgagggcggcggcccactcgcgggtcaaacccagaatcgcgcctttcgccgcagcgtaggcgctggtcttgccctgaccggtcaatgcggtcttggaaccgatattgaccaccgagcc   
